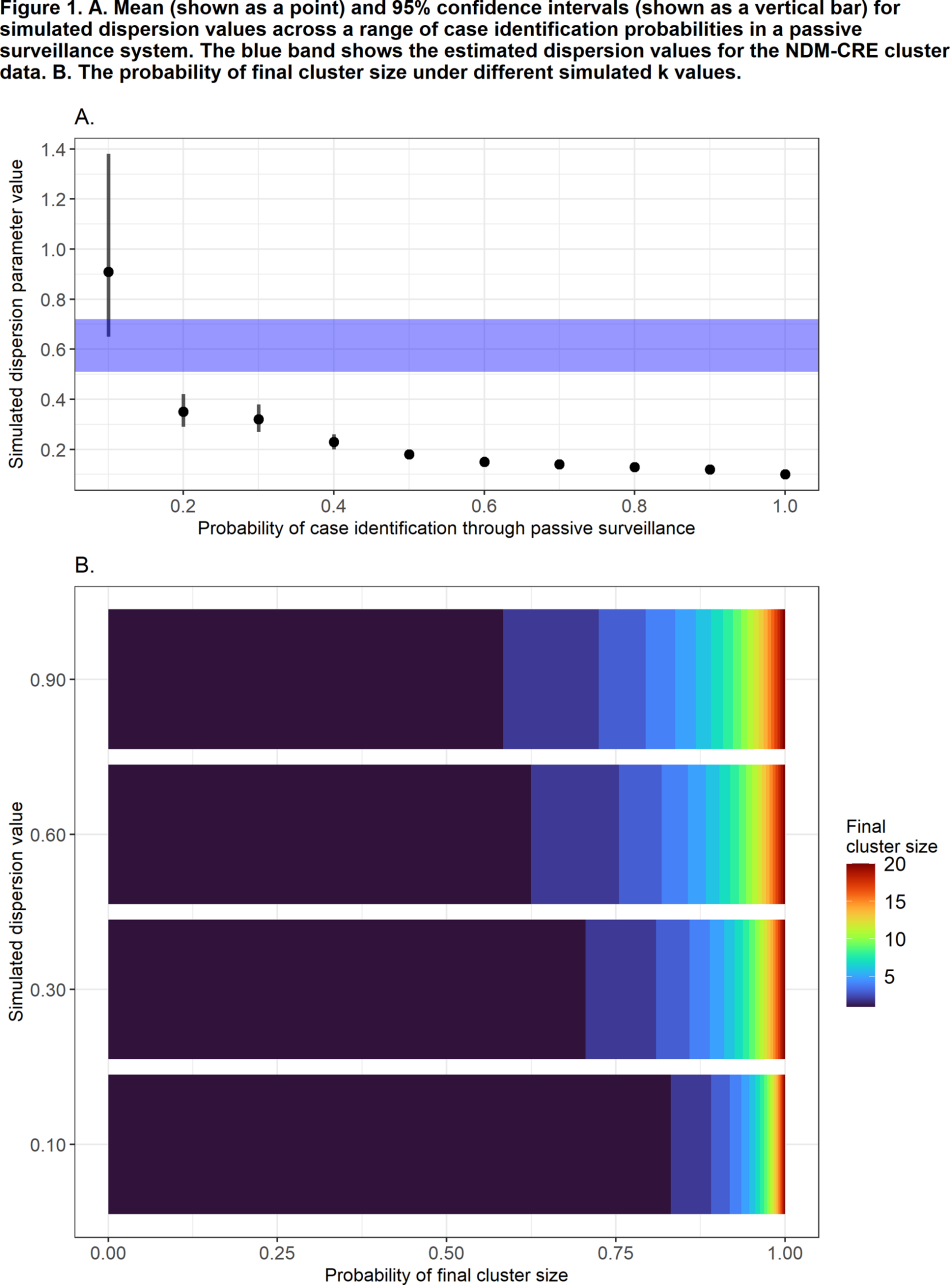# 331 Clinical Factors and Outcomes of Patients with Hospital Onset MRSA Bacteremia

**DOI:** 10.1017/ash.2026.10676

**Published:** 2026-06-23

**Authors:** Velma Lopez, Ashley Styczynski, Richard Stanton, Rachel Slayton

**Affiliations:** 1 Centers for Disease Control and Prevention; 2 CDC; 3 Centers for Disease Control and Prevention, Division of Healthcare Quality Promotion

## Abstract

**Introduction:** Transmission of New Delhi metallo-beta-lactamase-producing carbapenem-resistant Enterobacterales (NDM-CRE) is stochastic in nature. Largescale NDM-CRE outbreaks have been observed throughout the world. Within the United States (US), the incidence of NDM-CRE infections increased by 5-fold between 2019 and 2023, highlighting concern for large outbreaks. Given this, estimating how large an NDM-CRE outbreak is likely to be (i.e., the probability of final cluster size) is a valuable first step in characterizing NDM-CRE transmission dynamics and informing outbreak response. **Methods:** The National Center for Biotechnology Information (NCBI) Pathogen Detection database is a global, public tool that identifies clusters of bacterial pathogen genomes based solely on genetic relatedness. We included all NDM-CRE genomes associated with a US cluster and uploaded between January 2009 and September 2025. Using cluster data, we estimated the dispersion parameter (k), which reflects transmission chain variance. Because we assume that NCBI does not capture all NDM-CRE cases in a cluster, we compared the estimated k values for the NDM-CRE clusters to simulated k values. Our simulations varied the probability of case identification and assumed the average number of new cases from each infection was 1.1 (based on literature; see Figure 1A). Using these simulated k values, we estimated the probability of final cluster size (see Figure 1B). **Results:** We included 1,104 clusters with a mean size of 5 genomes (standard deviation=20). The estimated k value was comparable to, but higher than, simulated k values, supporting our assumption that NCBI cluster data are incomplete (Figure 1A). Comparing the probability of the final cluster size between estimated k values, which were likely biased (<0.3), to simulated values k values <=0.3, final cluster sizes are most likely to have two cases and a low probability of clusters <=10 cases (Figure 1B). **Conclusions:** Unlike many other infectious diseases, NDM-CRE cluster dispersion values remain unknown, which is a critical gap. We estimated dispersion to show that US NDM-CRE clusters have a high probability of being small. However, additional analysis is needed since standardized NCBI cluster data may be subject to biological, technical, and sampling limitations and do not allow for assessing why US clusters were small. Strong infection prevention and control practices and assessment of patient travel history continue to be best practices for mitigating NDM-CRE outbreaks. Moreover, as the NDM-CRE epidemiology continues to evolve, monitoring US NDM-CRE clusters will provide insight into outbreak dynamics, including emergence of highly successful clones.

**Figure:**